# The Evolution of Molecular Recognition: From Antibodies to Molecularly Imprinted Polymers (MIPs) as Artificial Counterpart

**DOI:** 10.3390/jfb13010012

**Published:** 2022-01-28

**Authors:** Ortensia Ilaria Parisi, Fabrizio Francomano, Marco Dattilo, Francesco Patitucci, Sabrina Prete, Fabio Amone, Francesco Puoci

**Affiliations:** 1 Department of Pharmacy, Health and Nutritional Sciences, University of Calabria, 87036 Rende (CS), Italy; ortensiailaria.parisi@unical.it (O.I.P.); fabriziofrancomano@libero.it (F.F.); marco.dattilo@unical.it (M.D.); francesco.patitucci@unical.it (F.P.); sabrina.prt@email.it (S.P.); 2 Macrofarm s.r.l., c/o Department of Pharmacy, Health and Nutritional Sciences, University of Calabria, 87036 Rende (CS), Italy; amonefabio@gmail.com

**Keywords:** molecular imprinting, molecularly imprinted polymers (MIPs), antibodies, synthetic antibodies, polymeric antibodies, plastic antibodies, theranostics, diagnostics, therapeutics, drug delivery systems (DDSs)

## Abstract

Molecular recognition is a useful property shared by various molecules, such as antibodies, aptamers and molecularly imprinted polymers (MIPs). It allows these molecules to be potentially involved in many applications including biological and pharmaceutical research, diagnostics, theranostics, therapy and drug delivery. Antibodies, naturally produced by plasma cells, have been exploited for this purpose, but they present noticeable drawbacks, above all production cost and time. Therefore, several research studies for similar applications have been carried out about MIPs and the main studies are reported in this review. MIPs, indeed, are more versatile and cost-effective than conventional antibodies, but the lack of toxicity studies and their scarce use for practical applications, make it that further investigations on this kind of molecules need to be conducted.

## 1. Introduction

Molecular recognition is a desired ability for molecules involved in various technological applications, in particular in the field of life sciences. Molecules with this property, indeed, are able to selectively bind compounds with biological importance (biomarkers such as microorganisms’ proteins and toxins, proteins and metabolites expressed in particular pathological conditions including cancer and autoimmune diseases), mainly for diagnostic, theranostic, pharmacological, drug delivery and research uses. Molecules of this type are present in nature, such as antibodies, which are normally produced by immune cells. Natural antibodies were extracted from cells and the production process was increasingly controlled and refined. Antibodies were used for many applications, but, in the last decades, different kinds of molecules of synthetic origin were designed to replace them.

This review is about molecules with molecular recognition capabilities, starting from antibodies and focusing on the more recent molecularly imprinted polymers (MIPs), deepening their design, synthesis and applications.

## 2. Antibodies

**Figure 1:**
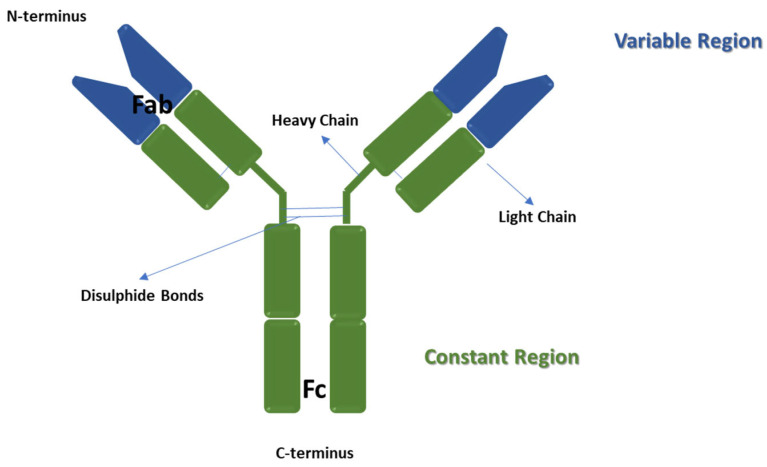
Schematic representation of the antibodies structure.

In 1891 Paul Ehrlich used the term *antibody* referring to proteins present in animal extracellular fluids (mainly in the blood) with an important role in specific immune responses [1]. Antibodies (also called immunoglobulins) are glycoproteins, whose primary role consists in the recognition of foreign substances (such as microorganisms components), called antigens, directly neutralising them or binding them for successive processing by other elements of the immune system [2]. These macromolecules, produced by plasma cells, which originate from B lymphocytes [3], are Y-shaped globular proteins formed by two light chains and two heavy chains, held together by disulphide bonds [4]. A schematic representation of antibodies structure is shown in Figure 1.

There are five classes of antibodies, which differ for the type of heavy chain and, consequently, for their function and localisation in the organism: IgA, IgD, IgE, IgG and IgM [5]. All the chains contain one N-terminal variable domain. The light chains are formed of one constant domain, whereas the heavy chains are made up of three or four constant domains. In detail, the heavy chains with three constant domains contain a hinge region between the first and the second domains [5]. An antibody contains the antigen-binding fragment (*Fab*) region, formed of the N-terminal end of both light and heavy chains, and the crystallisable fragment (*Fc*) region, which allows the interaction with the immune system cells or molecules [6].

**Figure 2:**
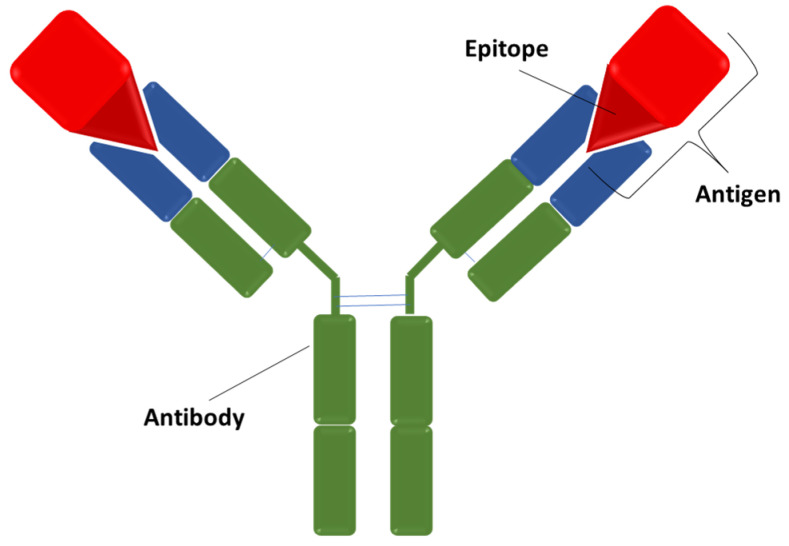
Interaction antigen–antibody. The ratio antigen/antibody is 2:1.

In 1952, Richard J. Goldberg described the interaction antigen–antibody (Figure 2), assessing that each antibody is capable to bind only to a specific antigen [7]. The interaction antigen–antibody is achieved between the epitope (a specific portion of the antigen) and the paratope (a specific portion of the antibody) [8]. Antigen and the relative antibody are bound through weak and non-covalent interactions, like Van der Waals forces, hydrogen bonds, hydrophobic and electrostatic interactions, resulting in a relatively stable bond [9]. The interaction between antigen and antibody is called agglutination and it is characterized by high specificity and affinity, resulting in the formation of an antigen–antibody complex that is a reversible reaction, hence there is a dynamic equilibrium between the complex and the dissociated form. The strength of the binding is expressed as avidity [10].

### 2.1. Antibodies Applications

The interaction antigen–antibody, or rather, molecular recognition made antibodies useful tools for many purposes. They are used for basic research, drug delivery, diagnostic, therapeutic and theranostic applications [11]. Antibodies (both monoclonal and polyclonal ones and their fragments) are daily used in cellular biology, molecular biology, biochemistry and medical research, in particular in the form of immunoassays, which are frequently employed for diagnostic use also, as well as for analytical applications in general [12]. In detail, antibodies or their fragments (single-domain antibodies or heavy chain-only antibodies) [13] are used in antibody conjugated signal generation systems for immunoassays. When an antibody binds to its target, label materials, linked to the macromolecules, emit a signal to quantify the entity of the binding of the antibody to its target. Label materials are usually radioactive isotopes [14] or enzymes, such as alkaline phosphatase and horseradish peroxidase [15], which form coloured, fluorescent or chemiluminescent products [16]. Examples of immunoassays are Western blotting [17], ELISA (enzyme-linked immunosorbent assay) [18], flow cytometry [19], surface enhanced Raman scattering (SERS) based immunoassay [20], Förster resonance energy transfer (FRET) immunoassays [21]. Furthermore, in immunoprecipitation, the measurement of the binding antibody-target is achieved directly on the complex, which precipitates [22]. In addition, lateral flow immunoassays (LFIAs), also called lateral flow immunochromatographic assays, are designed in order to detect the presence or absence of a specific analyte in the sample [23]. Immunoassays are used for the diagnosis of many diseases, retracing biomarkers, for example for cancer, but also other analytic purposes such as the detection of environmental pollutants and food contaminants [12].

Antibodies find application also in bioimaging techniques, such as magnetic resonance imaging (MRI), used, above all, for detecting cancer and fluorescence imaging [24]. Antibodies (or fragments) conjugates allow to enhance the ability of these techniques to discern between normal and cancer cells [25].

Furthermore, antibodies have been coupled to PCR (polymerase chain reaction) in order to increase the accuracy of this technique, avoiding the nonspecific amplification of undesired DNA sequences by Taq polymerase at low temperature [26] and, in the case of antibodies with enzymatic activities (called abzymes), to catalyse reactions [27]. 

In the last decades, theranostics has been developed from the combination of diagnosis and therapy [28,29]. Antibodies (or fragments) conjugated with nanomaterials have found many theranostic applications, in particular for cancer diagnosis and treatment [30], thanks to the ability to selectively accumulate in target tissues [31]. Theranostic antibodies have been developed for photoacoustic imaging and thermal ablation of gastric cancer cells [32] or for the imaging and treating of breast cancer cells [33]. Another example is provided by Webb et al. [34], who designed a conjugate for photothermal ablation of triple-negative breast cancer cells and SERS imaging of immunomarkers.

From a purely therapeutic point of view, the first monoclonal antibody with a therapeutic indication, approved in 1986, was the immunosuppressant muromonab-CD3 [35], which consists of a murine monoclonal antibody against T cell-expressed CD3 [36]. The first monoclonal antibody with an oncologic indication was rituximab, a chimeric anti-CD20 IgG1 approved for non-Hodgkin’s lymphoma in 1997 by US Food and Drug Administration (FDA) [35,37].

Polyclonal antibodies possess less suitable characteristics for therapeutic use, thus, their applications are limited to transplant rejection and few pathologies [38]. 

Nowadays, hundreds of monoclonal antibodies (or their fragments) and their conjugates have been studied in clinical trials and tens out of these have been approved as treatments for various diseases [35,39,40]:

- cancer (lymphoma, myeloma, melanoma, glioblastoma, neuroblastoma, sarcoma, colorectal, lung, breast, ovarian, head and neck cancers) [35,40,41];
- autoimmune diseases (arthritis, ankylosing spondylitis, psoriasis, multiple sclerosis, systemic lupus erythematosus) [42];
- inflammatory diseases (Crohn’s disease, asthma [43], Muckle–Wells syndrome [44], inflammations of the airways, skin and gastrointestinal tract) [35];
- infectious diseases (anthrax infection [45], prevention of Clostridium difficile infection recurrence [46], mycosis [47]);
- other conditions (migraine [48], transplant rejection [38], osteoporosis [49], high cholesterol plasmatic levels [50]).

Finally, antibodies have been physically or chemically [51,52] conjugated to nanosized drug delivery systems [53,54] due to their molecular recognition properties.

There are many studies in literature in which nanoparticles, often of magnetic type, linked to antibodies were preferentially accumulated in tumour cells than in the other tissues [55] resulting in a specific action of the active ingredients and avoiding their interaction with normal cells. 

### 2.2. Antibodies Production

Monoclonal antibodies are produced by plasma cells, are identical to each other and are able to recognize a specific antigen [35]. In 1975, Köhler and Milstein devised the hybridoma technique, which allows the quantitative production of pure monoclonal antibodies [56]. This technique consists in collecting spleen cells from mice that have been injected with the antigen, which triggers the immune response. After that, cells are fused to immortal myeloma cells originating the hybridoma, a cell line that indeterminately produces monoclonal antibodies against the specific antigen [56]. In order to increase monoclonal antibodies’ efficacy and duration, in the 1990s chimeric (by combining sequences of the murine variable domain with human constant region domain) [57] and humanized (through complementary-determining region grafting technique in which non-human antibody CDR sequences are transplanted into a human framework sequence in order to maintain target specificity) [58] antibodies were developed. Moreover, fully-human antibodies were produced through antibody phage display which is a genetic engineering technique in which RNA is collected from human B lymphocytes to create a cDNA library using reverse transcriptase enzyme. Then, the library is inserted in the bacteriophage, which infects bacteria that, in turn, produce human antibodies [59]. 

Polyclonal antibodies, instead, are produced by different cell lines, have a different amino acid sequence and recognize different epitopes of the same antigen. In order to produce polyclonal antibodies, animals are injected with the antigen; when the antibodies are formed, animal blood is extracted and purified to collect polyclonal antibodies [60]. Polyclonal antibodies are more stable and tolerant to changes in the antigen than the monoclonal ones and their production is easier and less expensive than the one necessary for preparing monoclonal antibodies, but several disadvantages need to be considered such as intra-batch variability, low specificity and a high degree of cross-reactivity [60].

However, both monoclonal and polyclonal antibodies need animals to be kept, which requires costs, care and qualified personnel, in order to be produced. Furthermore, the fact that it is necessary to wait for the animal immune response makes the production time longer.

### 2.3. Alternatives to Antibodies

Antibodies revealed themselves as very useful, but they have some limits, in particular monoclonal antibodies, which are the most used ones. The most relevant of these limits is the lack of reproducibility and the consequent antibody variability [61]. In fact, genetic drift is unavoidable after a long period of time spent from the first generation of hybridoma cells [62]. Moreover, monoclonal antibodies are rarely characterised and many preparations may be contaminated by nonspecific immunoglobulins from the host animal [63] requiring repeated validation for every new batch [64], particularly when antibodies are employed in methods that demand high sensitivity. Therefore, various alternative technologies with the same ability of molecular recognition, also called *antibody mimetics*, which are better in terms of properties, versatility and, above all, production costs and time, have been developed.

Aptamer technology is one of the main substitutes for antibodies. An aptamer is generally a molecule of nucleic acid (either DNA or RNA), but sometimes it can be a small peptide [65]. Aptamers are characterized by the same property as antibodies to bind molecules, mainly proteins, so these molecules are called *chemical antibodies* [62]. Aptamers were first isolated in 1990 and are chemically synthesized. In detail, they are generated in vitro following the SELEX (systematic evolution of ligands by exponential enrichment) method [66], which is a high throughput screening process. Aptamer sequence is defined, so, contrary to antibodies, a specific aptamer can be easily chemically reproduced avoiding variability issues [67]. Furthermore, the way in which aptamers are obtained is more cost-effective, rapid and easy to apply than antibody production. For all these reasons, aptamers may potentially be employed for all the applications in which antibodies are involved. In fact, nowadays, they could find applications in imaging, sensing, diagnostic, drug delivery, therapeutic, theranostic and analytical applications [68,69,70,71], even if this technology has still been less explored than antibodies and several challenges are associated with aptamers including cross-reactivity and poor specificity.

Other significant examples of substitutes of antibodies are provided by:

- affibodies (protein scaffolds), which are the result of protein engineering: they consist of a scaffold protein, made up of the IgG of a bacterial protein, on which a three-helix protein is inserted [62,72];
- RGD peptides, which are cyclic peptides formed of three amino acids, such as arginine, glycine and aspartic acid (RGD), and are used in drug delivery systems combined with active molecules for imaging [73], tissue repair [74] and tumour therapy [75], because of their ability to bind integrins in the extracellular matrix [74];
- small organic molecules, such as folic acid, which are conjugated to diagnostic and therapeutic agents in order to localise these molecules preferentially in cancer tissues, where the folic acid receptor is overexpressed [76].

Finally, molecularly imprinted polymers (MIPs) are the most studied antibody mimetics and the focus of this review [77].

## 3. Molecularly Imprinted Polymers (MIPs)

MIPs are polymeric matrices with the ability to selectively bind specific molecules. The molecular imprinting technique consists of the polymerization of monomers in the presence of a target molecule, which acts as a template during the synthesis of the polymer. The resulting products have an affinity similar to the affinity antibody–antigen, achieved through the formation of sites that are complementary to the template molecule. Molecular imprinting was first introduced in 1931 by Polyakov [78], who noticed that, when polymerization to get silica was conducted with benzene, the resulting silica showed unusual adsorption properties toward the additive. The scientist explained this evidence in terms of silica pore in polymeric structure. In 1949 Dickey [79] reported the same synthesis achieved by Polyakov, but the additional substance, in this case, was the dye methyl orange and the obtained silica was selective for this molecule. Dickey repeated the procedure using other dyes with similar results. After Dickey’s method, many chemists prepared specific silica-based sorbents with the same strategy; however, the interest in imprinted silica waned for the scarce stability and reproducibility of the materials. A great impulse to the development of molecular imprinting was given by Wulff and Sarhan [80], who, in 1972, introduced the covalent approach, and, above all, Mosbach, who, in the 1980s [81], devised the non-covalent approach.

To date, a lot of in vitro and in vivo studies have been carried out on MIPs, which will be deepened in the review.

### 3.1. MIPs Synthesis 

**Figure 3:**
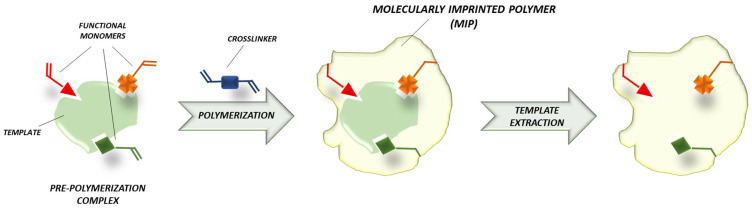
Molecular imprinting mechanism.

MIPs synthesis process (Figure 3) can be divided into three main phases:

- pre-polymerization, in which the template and the functional monomers form a complex;
- polymerization, in which monomers polymerize in the presence of the template and the cross-linking agent;
- the template removal [77].

The result is a stable polymer that is ready for the rebinding of the target molecule and whose characteristics (rigidity, porosity, molecular weight, resistance in different conditions and responsivity to various stimuli) can be modulated [77,82]. There are two main approaches in molecular imprinting relying on the nature of the interactions that form the pre-polymerization complex.

The covalent approach is based on reversible covalent bonds between template molecules and functional monomers. After polymerization, the template is removed from the polymeric matrix by chemical cleavage of the corresponding covalent bonds [77,83,84]. The greatest pro of the covalent approach lies in the strength of covalent interactions, determining a uniform distribution of the recognition sites and reducing the nonspecific interactions. The limits of this approach include the difficulty in obtaining a thermodynamic equilibrium [85], because of the strength of the covalent bonds, and, consequently, the possibility to recognize only a few target molecules [82], such as alcohols, aldehydes, ketones, amines and carboxylic acids [77].

The non-covalent approach, instead, is based on the formation of relatively weak non-covalent interactions between template and functional monomers, such as hydrogen bonds, ionic interactions, van der Waals forces and dipole–dipole interactions both during the polymerization and the rebinding phase [85]. This approach presents many advantages that make it preferable in comparison to the covalent one. In detail, non-covalent imprinting is characterized by the simplicity of the procedure and the possibility to use various monomers (methacrylic acid [86], 4-vinylbenzoic acid [87], acrylamide [88], vinyl pyrrolidone [89], 2-hydroxyethyl methacrylate [90], etc.) and to target a wide spectrum of molecules [77,91,92,93]. The disadvantages of the non-covalent approach are the request of a large excess of functional monomer, due to the need to shift the equilibrium toward the associated form of the pre-polymerization complex, and the formation of non-selective binding sites [77]. 

Starting from covalent and non-covalent approaches, Whitcombe devised the intermediate semi-covalent approach [94], in which the pre-polymerization interactions between the template and the functional monomers are covalent, whereas the rebinding step is characterized by non-covalent interactions. This approach allows to combine the high affinity in the formation of the pre-polymerization complex with the rapid binding kinetics in the rebinding phase [91,95].

**Figure 4:**
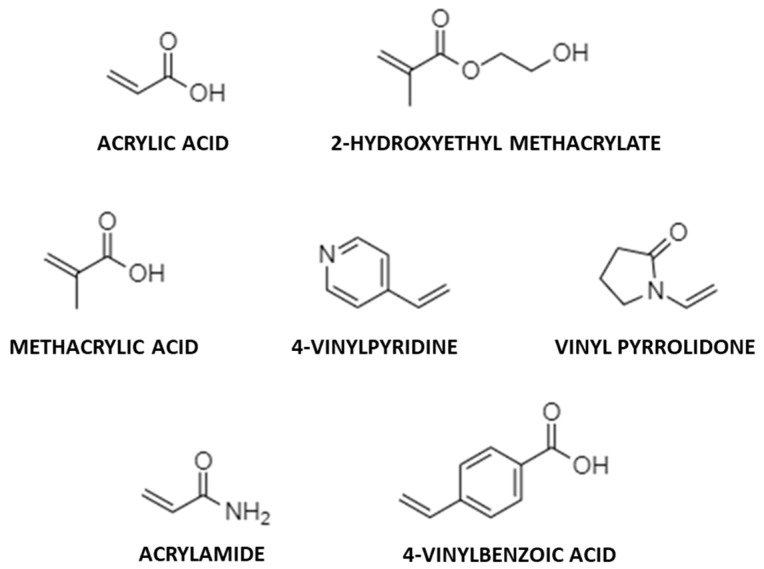
Chemical structure of some of the monomers used for MIPs synthesis.

There are many factors that need to be considered for the synthesis of MIPs such as the nature of functional monomers, crosslinking agents and initiators, the solvents, the ratio among the reagents, the polymerization technique and the imprinting approach [77,85]. The structures of some monomers used in molecular imprinting are reported in Figure 4.

Many polymerization techniques have been used for the synthesis of MIPs (Table 1).

The most classic method is free radical polymerization, which requires heat or light to activate the process [96]. Several monomers are suitable for this technique, but this strategy leads to the formation of high-branched, atactic MIPs with low binding specificity and selectivity [97], due to the inability to control propagation and termination phases, and a high polydispersity index [96]. This is the reason why controlled radical polymerization started to be preferred to free radical polymerization [98], because it is a more versatile group of reversible deactivation radical polymerization techniques (atom transfer radical polymerization [99], reversible addition-fragmentation chain transfer [100], nitroxide-mediated polymerization [101], iniferter-mediated polymerization [102]) that allow to control molecular weight distribution and stereochemistry, but not the size of the synthesized nanoparticles. Nowadays, the most frequently used techniques, specific for the synthesis of nanoMIPs, include precipitation polymerization, emulsion polymerization and core-shell polymerization with subsequent grafting [103,104,105,106].

**Figure 5:**
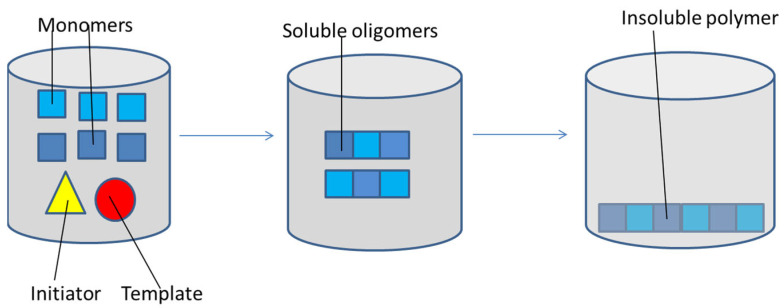
Precipitation polymerization: monomers, template and initiator are soluble; the chain grows until its size makes it insoluble, making the polymer precipitates.

Precipitation polymerization (Figure 5) was reported for the first time by Ye et al. in 1999 [107]. It is a simple method that allows the synthesis of uniform and spherical MIP nanoparticles in a single-step reaction. The method involves an excess of solvent, in which the monomers, the initiator and the template are soluble, whereas the polymer that will be formed is not. The formation of polymer chains from monomers and oligomers continues until their size makes them precipitate [103,108]. The obtained MIPs are collected by washing and centrifugation [109]. Important parameters to be modulated are solvent polarity, temperature and stirring. The pros of this technique are that it is not necessary to use any stabilizer molecules, whereas the cons are the need to use a high amount of template and the long time required [103,110].

**Figure 6:**
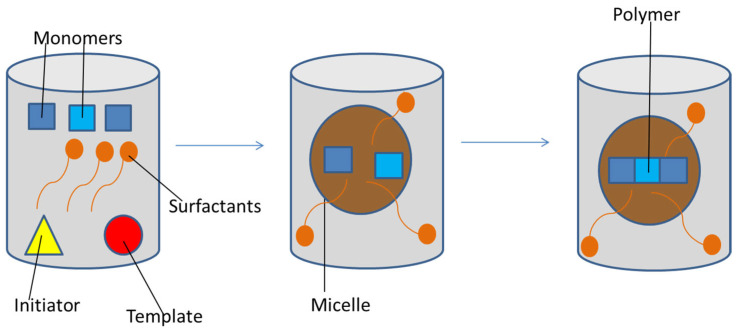
Emulsion polymerization: monomers are included in micelles with surfactants on their surface; the polymerization occurs inside the micelles.

Another important technique exploited for the production of nanoMIPs is emulsion polymerization (Figure 6) that enables the synthesis of monodispersed MIP nanoparticles [73] containing surface-exposed binding sites [111]. The polymerization process usually occurs in oil-in-water emulsions (less frequently also in water-in-oil emulsions) in the presence of a surfactant [112]. This technique can be performed in the form of mini- and micro-emulsion polymerization, obtaining nanoparticles of respectively 30–500 nm and 5–50 nm diameter [103]. A co-surfactant stabilizes the monomers, then homogenization is carried out by sonication or stirring [113]; the difference between mini- and micro-emulsion polymerization is that the latter requires higher surfactant concentration to get smaller particles. An advantage of emulsion polymerization is the high yield and the suitability to protein imprinting [73], but the obtained polymers often require purification, above all to remove the surfactant, with consequent waste of time [103].

**Figure 7:**
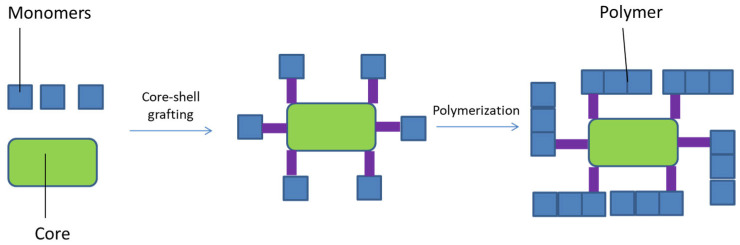
Core-shell grafting followed by polymerization.

Furthermore, a technique to produce core-shell MIP nanoparticles is core-shell grafting followed by polymerization (Figure 7) [103]. Preformed particles that compose the core, such as organic polymers [114], silica [115], superparamagnetic iron oxides [116], quantum dots [117], upconversion nanophosphors [118], carbon dots [119], and gold/silver particles [120], are linked to MIPs, which represent the shell of the nanoparticles. This technique allows fine control over MIP size [121].

**Figure 8:**
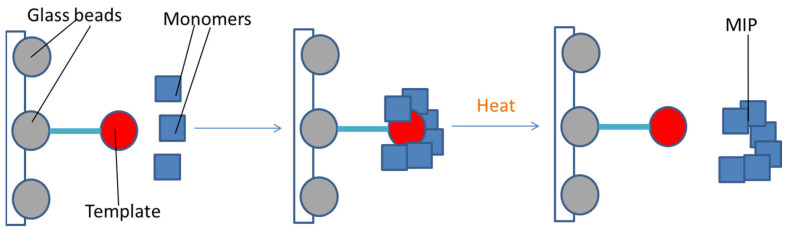
Solid-phase approach: the polymerization occurs around the template immobilized on glass beads; the detachment is obtained by heat.

The solid-phase approach (Figure 8) is one of the most recent devised techniques for nanoMIPs synthesis, which consists of the polymerization following the immobilization of the template molecule on glass beads activated in NaOH to expose -OH groups for the silanization [122]. After that, nanoMIPs need to be purified. With solid-phase synthesis, it is possible to synthesize MIPs with very high affinity and homogeneous distribution of the recognition sites, to recycle the template and avoid the washing phase for the removal of the template from the polymer [103,111]. On the other hand, the limits of this technique are the low yield and the fact that it is not very effective for templates with big structures [111]. 

Moreover, the detachment of the MIPs is conducted applying heat, which makes it impossible to increase the reaction yield by performing multiple syntheses on the same solid phase with thermosensitive templates like proteins [123].

Finally, high dilution polymerization is a method in which the monomer is dissolved in a high amount of solvent in order to avoid precipitation during the process [124]. This technique allows the synthesis of MIPs of a few nm, like natural antibodies size [125].

**Table 1:** Techniques for the synthesis of MIPs.

Technique (Ref.)	Mechanism	Pros	Cons
Free radical polymerization [96]	Free radical polymerization triggered by heat or light	Ease; wide choice of monomers	Low binding specificity and selectivity
Controlled radical polymerization [98,99,100,101,102]	Deactivation radical polymerization	Control of molecular weight distribution and stereochemistry	Lack of control of the nanoparticles size; high vulnerability to impurities and moisture; limited range of suitable monomers; not all vinyl monomers can be polymerized by ATRP (atom transfer radical polymerization); NMP (nitroxide-mediated radical polymerization) has been applied only once in the imprinting field due to the high required temperatures and the impossibility of using methacrylates
Precipitation polymerization [107]	The formation of polymer chains from monomers and oligomers continues until their size makes them precipitate	Uniform nanoparticles in a single-step reaction; need of a low amount of reagents required	Long time required; need of a high amount of template and solvent
Emulsion polymerization [73]	Polymerization in emulsions in the presence of a surfactant	High yield; suitability to protein imprinting	Required purification; use of a stabilizer
Core-shell grafting + polymerization [114,115,116,117,118,119,120,121]	Polymerization occurs around preformed nanoparticles	Control on MIPs size	Not effective for bulky templates
Solid-phase synthesis [122,123]	Polymerization follows the immobilization of the template molecule on glass beads	Very high affinity; homogeneous distribution of the recognition sites; recycle of the template	Low yield; not effective for thermosensitive and bulky templates
High dilution polymerization [124,125]	The monomer is dissolved in a high amount of solvent to avoid precipitation during the process	MIPs size is equal to a few nm	High amount of solvent

Moreover, surface-imprinted materials have recognition sites on the polymeric surface [110] and exhibit an enhanced selectivity and sensitivity [126] improving recognition effectiveness. They are produced by localizing the template on the polymeric surface [127] and the most frequently employed techniques are the solid-phase approach and emulsion polymerization [128,129].

A particular strategy, adopted when the template is a molecule difficult to obtain or to imprint due to the big size, is fragment imprinting, also called segment imprinting, which employs a portion of the target molecule as a pseudo-template to produce MIPs [130]. 

Dummy template imprinting is a method that uses substances similar to target molecules as templates for imprinting. This is useful when the templates are expensive or toxic molecules [131]. 

**Figure 9:**
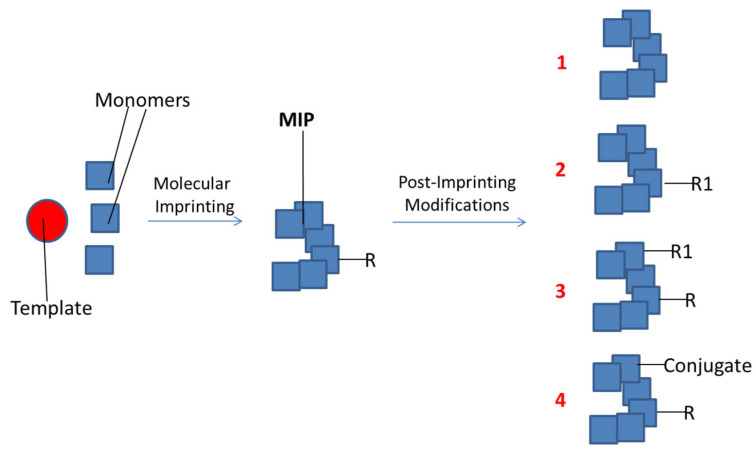
Post-imprinting modifications: (1) removal of a functional group; (2) transformation of a functional group; (3) addition of a new group; (4) conjugation with another molecule.

Like natural proteins after translation, MIPs can undergo post-imprinting chemical modifications [132], which consist of the addition of new functional groups, the transformation of functional groups and the conjugation with molecules (Figure 9) [133]. This approach is very useful to enhance the specificity and the sensitivity of MIPs. For this method, it is necessary that the functional monomers used for the polymerization contain functional groups that can be chemically modified, such as amino or carboxyl groups, or reversible bonds including disulphide and imine bonds. After molecular imprinting, it is possible to modify monomer residues with hydrolysis and other reaction mechanisms. Post-imprinting modifications allow to remove functional moieties that are undesired for MIPs applications or to introduce desired groups, without losing the selectivity and the specificity of molecular recognition. 

The site-specific introduction of fluorescent dyes is a very useful application of post-imprinting modifications. For example, Saeki et al. [134] reported a MIP for the detection of prostate-specific antigen (PSA). In detail, after the molecular imprinting phase, two post-imprinting modifications were made to the polymer. First, PIR-C, which is a new PIM (post-imprinting modification) reagent, was introduced reacting with polymer thiol groups and obtaining molecularly imprinted nanocavities with orthogonal dual interaction sites, in particular, 3-fluorophenyl boronic acid for the binding of PSA sugar chains and carboxyphenyl groups for PSA proteins linkage, fundamental for the specificity and selectivity of the interaction MIP-target. Then, the fluorescent dye Alexa Fluor 647 was introduced with the second post-imprinting modification on PIR-C secondary amino groups.

Furthermore, in order to avoid one of the limits of molecular imprinting, that is to say, the heterogeneity of the recognition sites in terms of affinity, another post-imprinting modification has been introduced: the capping. A good solution is the block of the functional monomer residues in the cavities with low affinity, followed by specific post-imprinting modifications of the residues in the high-affinity cavities [132]. A representative example is the development of a fluorescent MIP for lysozyme recognition. After the preparation of the lysozyme-imprinted polymer, a diluted lysozyme solution was added to the MIP film to protect the functional monomer residues in the high-affinity binding cavities. After that, the capping treatment was applied to the residues in the binding cavities with low affinity: p-isothiocyanatophenyl α-D-mannopyranoside reacted with the amino groups on the functional monomer moieties blocking them to the conjugation with the fluorescent molecule, which occurred instead in the high-affinity cavities. According to the experimental results, the capping significantly increased the MIPs selectivity [135].

### 3.2. Applications of MIPs as Antibodies

Molecular recognition is a fundamental MIPs property that, together with the fact that they are resistant in various chemical and physical conditions (pH, temperature, organic solvents, pressure) [77], makes these synthetic macromolecules potentially suitable for many applications.

#### 3.2.1. Sensors, Bioassays and Diagnostic Applications 

Classical bioassays, such as ELISA, are widespread, even if they are expensive and consist of long procedures [136]. Until MIPs were prepared through bulk polymerization, they were made up of microparticles [121], so they could not be used in bioassays due to the difficulty in immobilizing MIPs in microplate wells. When the techniques for obtaining MIP nanoparticles, such as solid-phase synthesis, were devised, MIPs acting as plastic antibodies (synthetic polymer nanoparticles with antibody-like functions as potential alternatives to protein antibodies [104]) started to be studied for pseudo-ELISA assays. For example, very sensitive assays for the detection of the antibiotics vancomycin [137] and gentamycin [138], cocaine [139] and bisphenol A (BPA) [140], containing MIP nanoparticles physically immobilized on the microplate surface, have been developed reducing production time and costs, with better stability that consents a long shelf-life [103]. In detail, a horseradish peroxidase (HRP)-template conjugate was used and the sensitivity was much higher than in other ELISA tests [121].

All these studies demonstrated that MIP nanoparticles, obtained by solid-phase synthesis, can find application in the development of pseudo-ELISA assays for the detection of target molecules in real samples and characterized by high selectivity and sensitivity and other advantages including the impact on time and cost of production cycles compared to traditional ELISA.

Other examples of MIPs for assay applications are the production of a MIP through sol-gel polymerization on the surface of microplates using recombinant human erythropoietin-α (rhEPO) as a template molecule [141], and the surfactant-mediated sol-gel polymerization in an aqueous environment designed in order to get MIPs for protein recognition [142]. Furthermore, new possibilities emerged using magnetic MIPs in microplates [143]. For example, magnetic nanoMIPs are employed in blood typing assays. MIPs were imprinted with blood type B trisaccharide [144] and the erythrocytes were removed from the solution by magnetic MIPs, causing decolourization. Antibodies used in biological sensors due to their high affinity and specificity, indeed, are vulnerable to denaturation when changes in temperature, pH and/or salinity occur. In these conditions, antibody-based blood typing tests might generate incorrect results. Therefore, the development of MIPs-based antibodies able to combine the selective recognition abilities of biological antibodies with the higher stability and low-cost production of the artificial counterpart was attractive as alternative to be employed in blood typing systems.

The molecularly imprinted polymer nanoparticles assay (MINA) was used for the analysis of biotin [145] and it exploits the competition of fluorescent nanoMIPs binding towards the free analyte and the analyte immobilized on magnetic inserts. The developed MINA allows to avoid the interference of other compounds, such as mercaptoethanol and sugars, that are well-known to affect traditional avidin and streptavidin-based assays. MINA has also been applied to the detection of the insecticide methyl parathion (MP) [146], proteins [147], leukotrienes and insulin [148] in biological samples. In particular, the developed MINA test for the detection of methyl parathion exhibited a high specificity and a linearity in a wide concentration range similar to ELISA. In addition, the assay allowed MP detection at picomolar concentrations without any cross-reactivity against other compounds such as chlorpyriphos and fenthion. Regarding MINA for leukotrienes and insulin detection, the assay showed comparable performance to existing chromatographic techniques, such as LC-MS/MS, and immunoassays in clinically relevant concentrations and using artificial urine and blood plasma. Therefore, MINA represents a very effective and versatile technique, which allows the use of stable reagents and the production of low-cost assays free of animal products. Other advantages are the fact that it can be applicable to many targets of clinical or environmental interest and to many kinds of matrix. Moreover, MIPs stability makes not necessary particular storage conditions and no washing steps or addition of enzyme substrates are required compared to the standard ELISA.

Furthermore, MIPs-based sensors have been devised, such as quartz crystal microbalance (QCM) and surface plasmon resonance (SPR) sensors, which require a high density of recognition sites close to the transducer [103]. For this application, MIP nanofilm is obtained through electropolymerization [149] or surface grafting [150]. Examples are the imprinted SPR sensors for the recognition of *Salmonella paratyphi* in contaminated water and food supplies, which provided a biosensing system characterized by good sensitive and selective responses [151], and QCM-based sensors for the recognition of tobacco mosaic virus also in complex matrices such as tobacco plant sap [152] and human rhinovirus antibodies [153]. Standard methods for the detection of microbial contaminants and the screening of viral infections, indeed, take a lot of time and effort due to the need for several experimental steps including sample preparation, cultivation, biochemical identification and serological confirmation. Therefore, the development of alternative and suitable control strategies attracts significant interest. 

One of the most recent examples of sensors is provided by Li et al. [154], who proposed an electropolymerization to obtain a system for the detection of deoxynivalenol (DON) by combining a highly-sensitive and selective sensor based on molecularly imprinted poly(L-arginine) with functionalized carbon nanotubes. 

A MIP-based electrochemical sensor for the detection of SARS-CoV-2 nucleoprotein has recently been developed [155]. The sensor is formed of a thin film electrode interfaced with a MIP synthesized to recognize SARS-CoV-2 nucleoprotein (ncovNP) and it is connected with a portable potentiostat. 

Moreover, a sandwich assay electrochemical biosensor was developed by You et al. [156] for the detection of amyloid-β oligomer (AßO), known as the biomarker of Alzheimer’s disease, through the formation of a sandwich composed of an aptamer and a MIP. In detail, core-shell nanoparticles, made up of silver and silica, presented the aptamer on their own surface. The described biosensor displayed high specificity, sensitivity, reproducibility and stability demonstrating the ability to detect AßO in human serum. Therefore, it could represent an innovative approach for the early diagnosis of Alzheimer’s disease characterized by several advantages including its low cost and the simplicity of the sample pre-treatment compared to natural antibodies employed in AβO detection.

The development of an electrochemical sensor with a MIP film for the detection of an antigen expressed in colorectal cancer (CEA, carcinoembryonic antigen), has been reported by Carneiro et al. [157]. The MIP layer was made up of polypyrrol, assembled in situ and combined with a poly(3,4-ethylenedioxythiophene) layer. The obtained sensor showed linear responses over a wide concentration range and high selectivity and reproducibility, thus, representing a very promising instrument to be used in CEA determination. 

Finally, a MIP obtained via the polymerization of a thiourea-based fluorescent functional monomer was obtained for the detection of non-steroidal anti-inflammatory drugs (NSAIDs), such as indomethacin and tolmetin [158]. Micromolar binding affinities were obtained in aqueous solution with performances similar to the ones observed for polyclonal antibodies. 

Table 2 reports a summary of MIP-based bioassays or sensors.

Desired characteristics for probes for bioimaging applications include the lack of toxic effects, biocompatibility and biodegradability or, if not possible, the possibility of rapid excretion [159]. MIP nanoparticles can be cleared through the kidneys if their hydrodynamic diameter is smaller than 8 nm, or through the liver. Other important characteristics for imaging are specificity, selectivity, affinity, size (preferably less than 100 nm), charge (cations have better reactivity with cells, whereas anions are less toxic) [111]. Parameters important to consider, specific for in vivo imaging, are biocompatibility, fluorescence (fluorophores need to emit in the near-infrared range to penetrate in deep tissues) and opsonisation, which needs to be avoided because immunoglobulins, in the blood stream, recognize nanoparticles as stranger elements and the immune system promotes their elimination. This last problem was solved by resorting to the coating of the MIP nanoparticles with a hydrophilic polymer layer of PEG [160] or albumin [161].

Bioimaging of glycans is fundamental to recognizing many pathological states such as cancer [111]. MIPs are important alternatives to antibodies and lectins, which form only weak interactions with polysaccharides [162]. NanoMIPs for bioimaging applications can easily be coupled with quantum dots [163] or fluorescent dyes [164] via surface functionalization.

A specific and selective MIP for the imaging of hyaluronic acid, a tumour biomarker, on epidermal keratinocytes was synthesized by precipitation polymerization [165]. For this purpose, D-glucuronic acid, which is a constituent of hyaluronic acid, was used as a template, (*N*-acrylamido)-benzamidine (AAB) and methacrylamide (MAM) were used as functional monomers and a polymerizable rhodamine derivative was employed as a fluorescent dye. This research study confirmed the applicability of fluorescently labelled MIP nanoparticles as artificial antibodies able to localize specific molecular targets, such as glycosylation sites on proteins, for cell and tissue imaging.

An example of MIPs-quantum dots for bioimaging is provided by Panagiotopoulou et al. [166], who devised a system based on quantum dots and core-shell MIPs made up of (4-acrylamidophenyl)(amino)methaniminium acetate and methacrylamide for the detection of cancer biomarkers D-glucuronic acid and N-acetylneuraminic acid, a component of sialic acid, on human keratinocytes at the same time. MIPs synthesized through photopolymerization, templated with D-glucuronic acid and coating carbon dots (CDs) [167] for the detection of hyaluronic acid in cancer and normal tissue are also present in literature. A poly(N-isopropylacrylamide)-based MIP, copolymerized with (4-acrylamidophenyl) (amino)methaniminium acetate and rhodamine B as functional and fluorescent monomers, respectively, was obtained by solid-phase synthesis [168] for the same purpose. Using this approach allows the synthesis of MIPs with the size of a few nm and homogeneous recognition sites. The difference is that an azide derivative of D-glucuronic acid was used as a template and immobilized on propargylated functionalized glass beads. 

Monosaccharides, such as mannose and fucose, are other cancer biomarkers, thus, many imaging techniques involving their recognition have recently been developed. For example, fluorescein isothiocyanate-doped silica nanoparticles, alternatively imprinted with fucose, mannose and sialic acid [111,125], were produced for specific and selective bioimaging and tested on human hepatoma carcinoma cells (HepG-2), normal hepatic cells (L-02), mammary cancer cells (MCF-7) and normal mammary epithelial cells (MCF-10A). Similar results were obtained with nanoMIPs associated with three quantum dots (emitting in the wavelengths corresponding to green, yellow and red) for multiplexed bioimaging [169]. Moreover, solid-phase is the best synthetic approach for bioimaging applications, because the fact that nanoMIPs are smaller allows the recognition of intracellular molecules, with results similar to the staining obtained with natural antibodies [111].

The latest studies described highlighted the high versatility and the potential for bioimaging of MIPs, which can be designed and prepared against a wide range of targets, from sugars and single amino acids to entire proteins. In addition, the combination of MIP materials with therapeutic agents and/or fluorescent/magnetic probes allows to obtain multimodal systems able to act as theranostic devices.

#### 3.2.2. Double-Imprinted Polymers as Targeted Drug Delivery Systems (TDDSs)

MIPs have been reported to be used for the controlled delivery of nutraceuticals [170,171] and therapeutic agents against cancer [172,173], when these compounds are employed as templates in the pre-polymerization stage, due to their high loading capacity and the good control exerted in the release process. In addition, MIP materials can also act as *targeted* drug delivery systems (TDDSs) able to recognize and bind specific cell markers and, thus, release the therapeutic agent to the target site avoiding side effects and improving patient compliance. In this case, the cell marker was used as a template while the drug can be loaded or conjugated to the polymeric material. 

Another very interesting approach to prepare MIP-based TDDSs is represented by double imprinting in which both the drug and the targeting agent are used simultaneously as templates. Double-imprinted polymers, indeed, are able to protect the therapeutic agent, prolong its release profile and, at the same time, to localize the drug delivery to the desired site improving the therapeutic profile and, thus, the therapeutic effects [174].

Double-imprinted MIP-coated mesoporous silica nanospheres were prepared using a conformational epitope of HER2 (Human epidermal growth factor) and doxorubicin as templates and dopamine as monomer. In detail, the MIP material was synthesized on the surface of silica nanoparticles according to the non-covalent imprinting approach and in vivo studies were carried out on human ovarian tumour-bearing mice showing that the drug concentration in the tumour was higher in the animal group treated with the developed doxorubicin-loaded delivery system [175]. 

In another study, doxorubicin was used as a template, together with a linear epitope of the epidermal growth factor receptor (EGFR) that is over-expressed in several tumours, to prepare a double-imprinted nanoMIP by solid-phase synthesis. The developed doxorubicin-loaded anti-EGFR nanoMIP was tested on different cancer cell lines and cytotoxicity and apoptosis were observed only in cells that over-expressed EGFR [176]. 

#### 3.2.3. Therapeutic Applications

The first therapeutic MIP, developed in 2010, was able to bind and neutralize the bee toxin melittin in mice blood reducing mice mortality [177]. The field in which MIPs have been more employed is cancer pharmacology and an important requirement for MIPs is a long retention time in the tumour tissue to deliver the drug [178].

A strategy for this purpose is the targeting of cadherins [111], proteins that promote cell–cell adhesion, whose uncontrolled expression favours cancer proliferation [179]. For this kind of application, an oligopeptide, a fragment of cadherin structure, was used as a template. The thermo-responsive MIPs were synthesized by solid-phase polymerization of N-isopropylacrilamide (NIPAM), N-tert-butylacrylamide (TBAM), N-phenylacrylamide (PAA) and 4-acrylamidophenyl (amino)-methaniminium acetate in the presence of the crosslinking agent with the oligopeptide immobilized on azide-functionalized glass beads [180]. The results of the study showed that MIPs were selective and significantly reduced the proliferation of tumour cells, more than monoclonal antibodies.

Another option for cancer therapy is the inhibition of the HER2 pathway [111]. In detail, HER2 is overexpressed in breast cancer [181] and exerts its action by dimerization with other proteins, like HER3. The inhibition of this interaction was previously achieved by monoclonal antibodies, but MIPs obtained via boronate affinity-oriented surface imprinting polymerization and conjugated with silica nanoparticles containing fluorescein isothiocyanate revealed themselves more effective in decreasing the cancer tissue in female mice [182].

Furthermore, MIPs are potentially applicable for the treatment of infectious pathologies because they can recognize and block components of etiological agents, such as bacteria, viruses, fungi and protista, involved in infection mechanisms. For example, Parisi et al. [183] used non-covalent imprinting to prepare a MIP able to recognize and bind the receptor-binding domain of the spike protein of SARS-CoV-2 (COVID-19 etiological agent), a surface glycoprotein involved in the recognition and the attachment to host cells [184]. The obtained imprinted nanoparticles were able to significantly inhibit virus replication in Vero cells culture. 

#### 3.2.4. Theranostic Applications

A growing number of theranostic applications of MIPs are emerging, exploiting the recognition properties of this kind of polymers for the simultaneous imaging and delivery of active molecules for the treatment and the ablation of cancer tissue by heat or reactive oxygen radicals [111]. In particular, nanoparticles seem to be the better mean for drug delivery in the whole organism [185,186]. Interesting characteristics of MIPs are the possibility of drug protection, reduced toxicity (especially for highly toxic anticancer drugs), controlled release and widespread distribution. Lately, MIPs have been used to target many membrane receptors, which are cancer biomarkers [118,187,188,189,190]. 

For example, MIPs selective for the epidermal growth factor receptor (EGFR) were synthesized by acrylamide reverse microemulsion polymerization in the presence of the crosslinking agent BIS and in association with red-emitting carbon dots [118].

In another study, a fluorescent MIP was designed for the delivery of doxorubicin. In detail, the MIP was made up of zinc acrylate, which interacted with the template through metal chelation, and acrylamide, which exploited hydrogen bonds and was synthesized via free radical polymerization and conjugated to fluorescent silicon nanoparticles [187]. In this case, the in vivo target was HER2, a glycoprotein overexpressed in breast tumour cells. In addition, the acidic environment in tumour tissues was able to break hydrogen bonds, thus releasing the drug specifically there, and the fluorescence allowed real-time monitoring of both MIPs and doxorubicin. 

MIPs composed of zinc acrylate, vinylphenylboronic acid and ethylene glycol dimethacrylate for the targeting of cancer cells in which Fn14 (fibroblast growth factor-inducible 14) is overexpressed, such as pancreas, brain and breast tumours and the delivery of the anticancer drug bleomycin were developed and tested in mice. The templates were the drug and a glycosylated epitope of Fn14, while silanized silicon nanoparticles with photoluminescence properties were used as the fluorescence source [188]. Bleomycin is released when the nanoparticles reach the cancer tissue with low pH, which leads to the breakage of the bond between vinylphenylboronic acid and the therapeutic agent. 

The folate receptor, overexpressed in various cancer cells, is another target of theranostic MIPs. In particular, MIPs, associated with fluorescein isothiocyanate linked to the nanoparticles and conjugated with poly(ethylene glycol)-folate, were obtained for precipitation polymerization in water using N-acryloyl-phenylalanine and N-acryloyl-lysine as functional monomers in the presence of the crosslinking agent N,N′-bis(acryloyl)cystamine [190]. This last forms disulphide bonds that are reduced by the big amount of glutathione in tumour cells causing the release of the anticancer drug vinblastine. The developed MIPs revealed themselves effective in xenograft mice injected with human cervical cancer cells.

Opsonisation can be circumvented by coating the MIP surface with polymers like polyethylene glycol, which prevent opsonins to bind to MIPs, avoiding their fast clearance [191]. Another strategy to overcome opsonisation is MIP synthesis using plasmatic albumin as a template, so that a layer made up of albumin proteins, present in the blood, is formed around the MIP. In this context, a theranostic MIP was obtained by precipitation polymerization of pyrrolidyl acrylate, NIPAM, 2-methacryloyloxyethyl phosphorylcholine, BIS and fluorescein acrylamide [192]. The prepared fluorescein-conjugated MIP was able to bind albumin, forming an albumin-rich protein corona, and to be passively accumulated in tumour tissue suggesting that this approach could be effective in the development of theranostic nanosystems for cancer therapy.

Photothermal therapy is used, coupled to imaging, to lead tumour cells to death causing localized hyperthermia [193] and, in this case, MIPs are very good substitutes of antibodies. For this purpose, plasmonic nanostructures have been exploited [194]. A system composed of gold nanorods, coupled with sialic acid-imprinted silica, was produced by a boronate affinity-oriented surface imprinting approach [119]. The formulation, containing also a fluorescent dye, is bound to the template resulting in the selective ablation of cancer cells overexpressing sialic acid. 

Another therapeutic technique, photodynamic therapy, based on the formation of a high amount of reactive oxygen species following light exposure on the target tissues, was associated with imaging. For the targeting of p32, MIPs able to recognize a conformational epitope of the extracellular N-terminal α-helix of the protein as template [195] were obtained through inverse microemulsion polymerization of acrylamide and BIS. The synthesized MIPs, incorporating the dye IR-783 and loading the photosensitizer methylene blue, were able to selectively recognize and stain p32-positive tumour cells, applying on them photodynamic therapy with no toxicity in the other tissues. 

For the targeting of folate receptor-α (FRα), MIPs, incorporating the dye IR-783 and loading the photosensitizer methylene blue, were synthesized through precipitation polymerization of acrylamide, 2-(trifluoromethyl)acrylic acid and BIS [196]. The template was an FRα N-terminal peptide forced to be organized in an α-helix structure. This formulation revealed itself able to localize the staining and the photodynamic effect on tumour cells overexpressing folate receptors. 

With a similar strategy, Fn14 was targeted [197]. MIPs made up of acrylamide, BIS, TBAM and APMA, incorporating the dye IR-783 and loading the photosensitizer hypocrellin B, were synthesized using a transmembrane helical portion of Fn14 as a template. The obtained nanoparticles showed a good in vivo antitumor activity in mice upon laser activation and no cytotoxicity in sane tissues. 

A MIP-based theranostic system, delivering the anticancer drug Sunitinib and incorporating the fluorescent marker Rhodamine 6G, was recently devised. The synthetic strategy consisted of the precipitation polymerization of methacrylic acid and ethylene glycol dimethacrylate in the presence of the therapeutic agent, followed by the functionalization of the obtained MIPs with the dye through radical grafting [198]. 

### 3.3. Comparison between MIPs, Antibodies and Aptamers 

Monoclonal antibodies and MIPs, due to their ability of molecular recognition, may have similar applications, with comparable affinity. Antibodies, in particular monoclonal antibodies, have poor chemical stability, reducing the shelf life of antibodies-based products and requiring a constant cold chain supply [103]. Instead, MIPs are stable in several environments, allowing the reuse of them for successive applications. In detail, MIPs are resistant to the variation of pH, temperature and pressure and to the exposure to organic solvents [77]. 

Furthermore, as previously described, monoclonal antibodies production involves animals, with all the disadvantages that this determines, such as the unseemly production cost and time (months), as well as the need for specialized personnel [103]. One of MIPs advantages toward antibodies lies in the low cost for both the production process and the research and development phase for new formulations, with shorter production time (few weeks) [121]. Molecular imprinting technology is more versatile because of the possibility to choose among thousands of monomers, and MIPs are able to recognize a wider spectrum of molecules than monoclonal antibodies can. MIPs avoid also the typical problem of antibodies of the batch-to-batch variability and they have the useful possibility to be conjugated easily to other molecules.

Although MIPs present so many potential advantages towards the antibodies, many questions continue to be on these molecules, above all the lack of studies about their toxicity, immunogenicity and clearance and the absence of massive use of MIPs in clinical and research practice [103].

Moreover, from the comparison between MIPs and aptamers, it emerges that they have many common features: high affinity for targets, high thermal stability, production mode, a wide range of target molecules, the possibility of functionalisation, low batch-to-batch variability [121]. The main differences lie in the lower stability to pH and organic solvents of aptamers, the higher cost and time production and the limited possibility to choose monomers (four nitrogen bases).

Table 3 points out the main differences among antibodies, aptamers and MIPs.

## 4. Conclusions

This review focused on molecules with the capability to recognize specific targets. Antibodies naturally have this property and they have found a lot of applications in many fields: drug delivery, immunoassays and biosensors for diagnostics, immunotherapy, theranostic, biological and pharmacological research. Some limits of this kind of molecules, like low stability, high cost and immunogenicity, made necessary the research of other solutions, like aptamers and, above all, molecularly imprinted polymers. These last ones, although nowadays have not had great commercial success, showed many potential advantages toward their biological counterparts, such as low cost, versatility and stability.

For all these properties, it is worth investing further resources in MIPs research, in particular, to translate the existing knowledge of practical applications and evaluate immunogenicity, toxicity and clearance, which could be a limit for their usage.

## Figures and Tables

**Table 2:** Some examples of MIP-based bioassays and sensors.

Bioassay or Sensor	Synthesis Technique	Targets	Key Findings	References
Pseudo-ELISA	Solid-phase approach	BPA, vancomycin, gentamycin, cocaine	Sensitivity better than previously described antibodies-based ELISA; low cross-reactivity	[137,138,139,140]
Blood typing assays	Solid-phase approach	Blood antigens	First example of preparation of MIPs for the selective recognition of a trisaccharide and potential application for diagnostic purposes; an effective alternative based on MIP materials to the use of conventional antibodies in the blood typing assays	[144]
Molecularly imprinted polymer nanoparticles assay (MINA)	Solid-phase approach	Biotin, methyl parathion, proteins, leukotrienes, insulin	High stability during storage also without refrigeration; no susceptibility to other molecules able to affect avidin and streptavidin-based assays; no cross-reactivity	[145,146,147,148]
MIP-based SPR and QCM sensors	Electropolymerization, surface grafting	Salmonella paratyphi, tobacco mosaic virus, human rhinovirus antibodies	Selective and sensitive MIP-based SPR biosensors for monitoring of microbial contaminants; monitoring of plant viruses directly in the plant sap within minutes; plastic copies of antibodies synthesized by imprinting procedures lead to very promising QCM systems for virus sensing	[151,152,153]
Electrochemical biosensors	Electropolymerization	Amyloid-β oligomer, colorectal cancer marker	High specificity, sensitivity, reproducibility and stability	[156,157]
Fluorescent sensing	Free-radical Polymerization	NSAIDs	Performances similar to the ones observed for polyclonal antibodies	[158]

**Table 3:** Main differences among antibodies, MIPs and aptamers.

Characteristic	Antibodies	MIPs	Aptamers
Thermal stability	Low	High	High
pH stability	Low	High	Low
Organic solvent stability	Low	High	Low
Immunogenicity	High	-	Low
Production cost	High	Low	Medium
Production time	>6 months	Few weeks	Few months
Production method	Animal immunization	Chemical synthesis	Chemical synthesis
Batch-to-batch variability	High	Low	Low
Amount of usable monomers	About 20 (amino acids)	Thousands	4 (nitrogen bases)
Amount of target molecules	Medium	High	High
Possibility of functionalization	Low	High	High
Amount of practical applications	High	Low	Medium
